# Neutrophil extracellular traps and neutrophilic dermatosis: an update review

**DOI:** 10.1038/s41420-023-01787-2

**Published:** 2024-01-10

**Authors:** Sheng Li, Shuni Ying, Yuqian Wang, Yelu Lv, Jianjun Qiao, Hong Fang

**Affiliations:** Department of Dermatology, The First Affiliated Hospital, Zhejiang University School of Medicine, Zhejiang, China

**Keywords:** Mechanisms of disease, Interleukins

## Abstract

Neutrophils have both antimicrobial ability and pathogenic effect in the immune system, neutrophil extracellular traps (NETs) formation is one of the representative behaviors of their dual role. NETs formation was triggered by pathogen-related components and pathogen non-related proteins as cytokines to exert its effector functions. Recent studies indicate that the pathogenicity of NETs contributed to several skin diseases such as psoriasis, Stevens-Johnson syndrome, toxic epidermal necrolysis, and neutrophilic dermatosis. Especially in neutrophilic dermatosis, a heterogeneous group of inflammatory skin disorders characterized with sterile neutrophilic infiltrate on dermis, NETs formation was reported as the way of participation of neutrophils in the pathogenesis of these diseases. In this review, we describe the different processes of NETs formation, then summarized the most recent updates about the pathogenesis of neutrophilic dermatosis and the participation of NETs, including pyoderma gangrenosum and PAPA syndrome, Behçet syndrome, hidradenitis suppurativa, Sweet Syndrome, pustular dermatosis and other neutrophilic dermatosis. Furthermore, we discuss the link between NETs formation and the development of neutrophilic dermatosis.

## Facts

- Neutrophil extracellular traps have been reported in some skin diseases.
- Aberrant neutrophil activation is crucial in the development of neutrophilic dermatoses.
- Neutrophil extracellular traps play a crucial role in the pathogenesis of neutrophilic dermatoses.

## Open Questions

- How do neutrophils generate neutrophil extracellular traps?
- What role does neutrophils play in the pathogenesis of neutrophilic dermatoses?
- Are neutrophil extracellular traps considered a pivotal factor in the pathogenesis of neutrophilic dermatoses?
- What is the regulatory mechanism underlying the generation of neutrophil extracellular traps in neutrophilic dermatoses?

## Introduction

Neutrophils are the main innate immune effectors in human defense system. In physiologic conditions, neutrophils exit in peripheral blood as the first line of antimicrobial system [1]. After septic or aseptic injury, abundant neutrophils released from the bone marrow into the circulation and compromised tissue [2]. Lower neutrophil blood counts caused by impaired maturation of neutrophil granulocytes lead to recurrent and life-threatening infections beginning after birth [3], which indicate that neutrophils play a vital role in human homeostasis. In other ways, however, neutrophils can also initiate and exacerbate life-threatening diseases like Stevens-Johnson syndrome and toxic epidermal necrolysis [4], play the harmful role in human system. As a double-edged sword for immune system, neutrophils have antimicrobial ability and pathogenic effect after inappropriate activation.

Activated neutrophils work through several ways to exert their effector functions, including phagocytosis, degranulation, and neutrophil extracellular traps (NETs) formation [5]. The release of NETs occurs through a cell death process named NETosis [6]. It can be triggered by a variety of agents such as pathogens, cytokines, pathogen-associated molecular pattern molecules, damage-associated molecular patterns molecules, immune complexes [7]. In humans, two heterogenous groups of neutrophils have been reported: typical polymorphonuclear neutrophils (PMNs) and low-density granulocytes (LDGs) found in individuals suffering from autoimmunity [8]. As LDGs reported with higher staining for neutrophil elastase (NE) and lower staining for secretory leukocyte proteinase inhibitor (SLPI, an inhibitor for NE) in contrast to PMNs, LDGs are more prone to occur NETosis [9]. In addition to neutrophils, eosinophils, macrophages, mast cells, and basophils have all reported to release extracellular DNA to form different DNA trap types [10–14].

Upon septic or aseptic conditions, the dysregulation of NETs release can cause damage in multi-system in humans. NETs was reported in the pathophysiology of several conditions including infection, sepsis, cancer, thrombosis and connective tissue diseases [15]. With regard to dermatological diseases, the research on NETosis was involved in psoriasis, pustular dermatosis and neutrophilic dermatosis [16, 17]. Especially in neutrophilic dermatosis, a recent study has revealed the role of neutrophils in autoinflammation and tissue damage in these diseases. Therefore, we sought to update the effect of NETs in the pathogenesis of neutrophilic dermatosis.

## Mechanisms of NETs formation

**Fig. 1:**
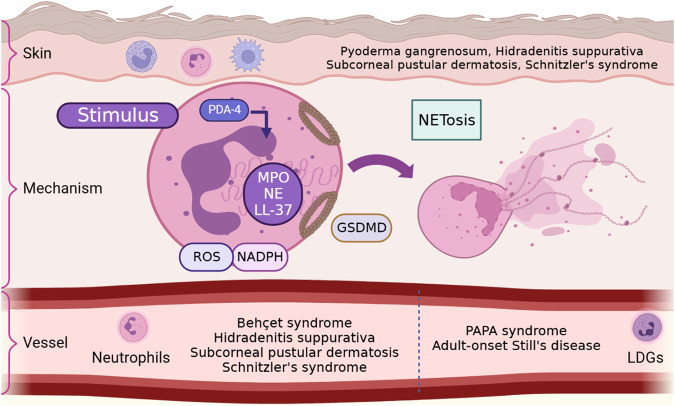
The formation of neutrophil extracellular traps (Created with Biorender.com). After being stimulated by external factors, neutrophils activate NADPH to release ROS. The activation of NADPH and ROS leads to the secretion of PAD4, which acts on eosinophilic granules and induces the release of proteins such as NE and MPO. These granular proteins will cause chromatin decondensation, and the secreted PAD4 will lead to histone citrullination. After the neutrophil plasma membrane ruptures, the decondensed chromatin combines with the granular proteins and is released to form a network-like DNA structure extracellularly. GSDMD is involved in the formation of eosinophilic granules and membrane pores. There are two types of neutrophils that can produce NETs, PMNs and LDGs. The production of NETs by neutrophils is involved in the occurrence of various skin diseases, such as pyoderma gangrenosum, PAPA syndrome, Behcet’s syndrome, pyogenic hidradenitis, adult Still’s disease, subcorneal pustular dermatosis, and Schnitzler’s syndrome. NADPH: nicotinamide adenine dinucleotide phosphate, ROS: reactive oxygen species, PAD: peptidylarginine deiminase, NE: neutrophil elastase, MPO: myeloperoxidase, DNA: deoxyribonucleic acid, PMNs: polymorphonuclear neutrophils, LDGs: low-density granulocytes, NETs: neutrophil extracellular traps, GSDMD: gasdermin D.

NETs formation is one of the major ways for neutrophils to exert their dual physiological functions described above. Appropriate NETs release is the first-line immune response to assist human body resist the invasion of external stimuli, while dysregulated NETs will lead to a variety of immune diseases [7]. The concept of NETs was first put forward by Takei et al. in 1996 and the systematic research of NETs was reported by Brinkmann et al. in 2004 [18, 19]. In general, the NET formation involves the extrude large amounts of intracellular contents accompanied with cell death and termed this process with NETosis (Fig. 1) [6]. According to the survival status of neutrophils, the process of NETs formation is divided into two types: lytic form of NETosis with the death of neutrophils and non-lytic form of NETosis with survival of neutrophils [19–21]. Although the non-lytic form of NETosis is considered should not be called as NETosis based on its definition [22].

NETs formation can be initiated by various factors, including microorganisms such as bacteria, fungi and their products like cytokines, chemokines and oxidized mitochondrial DNA [23–26]. In the initial of cell-death-dependent NETosis, neutrophils were stimulated with phorbol 12-myristate 13-acetate (PMA), which activates protein kinase C (PKC) and lead to the generation of reactive oxygen species (ROS) with nicotinamide adenine dinucleotide phosphate (NADPH) oxidase [27]. Upon activation of NADPH oxidase and ROS, the secretion of PAD4 is facilitated, acting upon eosinophilic granules, thereby promoting the release of proteins such as NE and MPO. Activated myeloperoxidase (MPO) and NE from azurophilic granules by ROS dislocated into the nucleus, leading to the disconnection of histones with chromatin, resulting in chromatin decondensation [19, 27]. In addition, immune complexes such as nicotine can trigger NETosis independently of NADPH oxidase [28], but still accompanied with cell death, named NADPH oxidase-independent, mitochondrial ROS-dependent NETosis [26]. Besides, NETs formation generated with non-lytic form when exposure to bacteria such as S. aureus [29]. The chromatin material released from live cells using an intact plasma membrane through intracellular vesicles [30].

In addition to the processes mentioned above, calcium channels on the cell membrane also participate in NETs production. Studies have shown that the opening of calcium channels on the cell membrane is necessary for raising intracellular calcium levels and triggering NETosis after neutrophil activation. It has also been demonstrated that reducing the concentration of extracellular free calcium can effectively inhibit NETs production, which further confirms the participation of calcium channels [31–34]. Blocking receptors such as Toll-like receptors (TLR) 2, cluster of differentiation (CD) 18, and G protein-coupled receptors (GPCRs) can also inhibit the generation of NETs in response to corresponding stimuli, indicating the involvement of TLR2, CD18, and GPCRs in NETs production [20, 33, 35]. Additionally, Janus kinase (JAK) 2 is also involved in the process of NETs formation [36, 37].

Gasdermin D (GSDMD) is also involved in NETs formation. Chen et al. found in 2018 that GSDMD can induce NETosis in neutrophils, leading to cell death, which is a novel form of GSDMD-mediated cell death [38]. Subsequently, Silva et al. found that blocking GSDMD can reduce NETs and alleviate symptoms in a septic mouse model [39]. In later studies, Silva et al. also found that GSDMD-mediated NETs formation is involved in tissue damage caused by COVID-19 [40]. The mechanism by which GSDMD mediates NETs formation may be that the pores formed by GSDMD promote the release of NE and MPO from azurophilic granules, while NE can reactivate GSDMD formation in a loop, and GSDMD also participates in the formation of membrane pores on neutrophils, releasing chromatin and bound granule proteins through the pores.

## Neutrophilic dermatosis

### Pyoderma gangrenosum and pyogenic arthritis, PG and acne (PAPA) syndrome

Pyoderma gangrenosum (PG) is a typical neutrophilic dermatosis characterized by painful skin ulcers with undermined borders and peripheral erythema [41]. The frequency of second immune-related systemic diseases in PG patients was 33% to 56.8% [42, 43], including inflammatory bowel disease, inflammatory arthritis and hematological malignancies. The most recognized inducer of disease is trauma, which causes the dysregulation of inflammatory cytokines and immune responses. The release of cytokines like IL-36 leading to the recruitment of neutrophils, cause the upregulated expression of neutrophil-attracting cytokines like IL-8, finally result in tissue damage [44].

One guess about how neutrophils produce its marked effect is through NETs formation. NETs formation was first reported in the PG skin lesion samples when compared to Schnitzler’s syndrome as a positive control [45]. This manifestation was further confirmed in recent studies [46, 47], one of them concluded 17 PG cutaneous lesions, with 62.9% neutrophils showing NETs, higher than different entities of neutrophilic dermatoses including Sweet’s syndrome and subcorneal pustular dermatosis [46].

PAPA syndrome is one of syndromic PG, which belongs to the autosomal dominant disorder. The study of PAPA syndrome revealed the elevated circulating LDGs and these LDGs displayed enhanced NETs formation and impaired NETs degradation. The research also indicated the effect of IL-1 signaling in exacerbated neutrophil responses in PG patients [48].

### Behçet syndrome

Behçet syndrome is a systemic vasculitis that affects both small and large veins and arteries, involving the multi-organs including skin, mucosa, eyes, etc. [49]. The pathophysiology of Behçet syndrome is complicated, with a strong contribution of genetic factors that almost half of patients carry the allele HLA-B*51 [50, 51]. As a result, some researchers preferred Behçet syndrome to the group of MHC-I-opathy disease with T lymphocyte-mediated immune dysfunction [52–54]. Besides, Behçet syndrome is also an autoinflammatory syndromes with neutrophil hyperactivation [16]. It is very important to reveal the mechanism of infiltrated neutrophils in circulation and other organs.

Circulating Neutrophils from patients with Behçet syndrome were more prone to release spontaneous or stimulated NETosis compared healthy control, with higher level of soluble CD40 ligand, peptidylarginine deiminase 4 and interleukin-17 [55–57]. Markers of NETs levels including cell-free DNA and myeloperoxidase-DNA were elevated in Behçet syndrome patients and DNAse treatment significantly decreased thrombin generation in Behçet syndrome plasm, indicated that inhibition of NETs may represent as a potential therapeutic target for Behçet syndrome [58]. Besides, macrophages stimulated with Behçet syndrome-derived NETs produced higher levels of IL-8 and TNF-α compared to heathy controls, and promoted IFN-γ CD4 T cells differentiation [59]. Conversely, morning saliva from patients with Behçet syndrome prone to oral ulcers failed to induce NETosis, it may demonstrate the different disordered homeostasis in the oral cavity [60].

### Hidradenitis suppurativa

Hidradenitis suppurativa is an inflammatory disorder that manifested with painful nodules, abscesses and sinus tracts [61]. The disease usually occurs in adulthood and last for a long time, and affect the patients both in physical and mental health because of its severe pain and bad smell [62, 63]. The initial part of disease onset is immune cells activation induced by microbial components and danger-associated molecular patterns (DAMPs). These immune responses lead to the release of cytokines including IL-1β and tumor necrosis factor (TNF) [64], then induce the production of chemokines such as CXCL8, CXCL11, CCL2 and CCL20 in keratinocytes and CXCL1 and CXCL6 in fibroblasts [65, 66]. Dysregulated cytokines and chemokines contribute together to the infiltration of neutrophils, T cells, B cells, monocytes and developed the disease [67].

The prominent presence of NETs was found in the hidradenitis suppurativa lesions and coexisted with plasmacytoid dendritic cells, in association with a type I interferon (IFN) gene signature. Also, the NETs were correlated with disease severity. Circulating neutrophils also manifested enhanced spontaneous NETs formation compared to healthy control neutrophils [68]. Besides, the increased formation of NETs was associated with tunnels according to the immunohistochemistry of hidradenitis suppurativa biopsy specimens [69]. To find the association between microbial components and NETs, the result of the RNA gene amplicon sequencing showed that Finegoldia magna was overabundant in skin samples and derived local inflammation to promote the formation of NETs [70, 71].

### Pustular dermatosis

Pustular dermatoses are a group of skin diseases characterized by aseptic pustules of varying sizes, with the pathological feature of massive neutrophil infiltration in the pustules [16]. Pustular skin diseases include generalized pustular psoriasis (GPP), impetigo herpetiformis (IH), acute generalized exanthematous pustulosis (AGEP), and subcorneal pustular dermatosis (SPD) [72–75]. The onset of these diseases is related to neutrophil infiltration, but the specific form of neutrophil activation and regulation mechanism are still unclear.

Research has demonstrated that compared with HC, there is a significant increase in the proportion of neutrophils producing NETs in the skin lesions of patients with SPD. This suggests a potential involvement of NETs in the pathogenesis of SPD [46]. IL-36 and its receptor antagonist (IL-36Ra) are crucial in maintaining physiological homeostasis [76–78]. Loss-of-function mutations in IL36RN, which encodes IL-36Ra, lead to a dominantly inherited autoimmune inflammatory disease termed Deficiency of Interleukin-36 Receptor Antagonist (DITRA) [79]. Using imiquimod cream to construct a psoriasis mouse model in IL-36Ra knockout mice, researchers found that the epidermis significantly proliferates, the number of neutrophils increases, and NETs expression significantly increases, indicating the involvement of IL-36 in NETs formation and the pathogenesis of psoriasis [80]. MPO, similar to IL-36, also plays a crucial role in neutrophil NETs formation and other physiological functions [81, 82]. Haskamp et al. discovered that mutations in MPO genes in 31 GPP patients affected neutrophils and monocytes, which results in reducing the induction of NETs formation in MPO-deficient neutrophils. Furthermore, CD47 expression is increased in MPO-deficient neutrophils. CD47 is an inhibitory protein for monocyte-mediated phagocytosis. The elevated CD47 expression can inhibit the phagocytosis of neutrophils by monocytes, thereby prolonging the survival time of neutrophils [83].

### Other neutrophilic dermatoses

Other neutrophilic dermatoses also showed the formation of NETs in skin lesions and circulation, including Schnitzler’s syndrome and adult-onset Still’s disease (AOSD).

Schnitzler’s syndrome is a rare autoinflammatory disorder characterized by neutrophil-dominated inflammation, which is induced by IL-1β [84]. Immunofluorescence co-staining showed widespread and substantial NETs formation in lesion skin of Schnitzler’s syndrome patients compared to the control skin. Blood neutrophils from patients showed significantly elevated NETosis rates compared to control neutrophils following stimulation with PMA [45].

AOSD is a rare systemic autoimmune inflammatory disease with an unknown etiology [85]. The disease is characterized by recurrent high fever, arthritis and joint pain, transient skin rash, leukocytosis, and hyperferritinemia [86–88]. The pathogenesis of AOSD remains uncertain, and multiple factors, such as infection, genetics, and immune dysfunction, may contribute to the onset of the disease. Macrophages and neutrophils, in addition to the cytokines released after their activation, play a crucial role in the pathogenesis of AOSD. Various external stimuli, such as DAMPs, can activate the inflammasome and ultimately cause dysregulated cytokine secretion by increasing the expression levels of IL-1β and IL-18, leading to cytokine storms and disease onset [89–92]. Additionally, the inflammasome pathway also participates in the pathogenesis of AOSD and its complications, macrophage activation syndrome, by mediating cytokine secretion through GSDMD [93]. Studies have found that AOSD patients have significantly increased levels of cell-free DNA, NET-DNA complexes, and α-defensin in circulation, as well as NE-positive and MPO-positive neutrophils in AOSD skin lesions compared to HC. Furthermore, these indicators are positively correlated with disease severity, and AOSD patients have a significantly enhanced ability of neutrophils to spontaneously produce NETs [89, 94, 95]. The number of LDGs and the expression levels of NETs are significantly higher in active AOSD patients, and the serum levels of NETs are positively correlated with the number of joint swelling and monocyte count [96]. Research has also found that type I interferons can trigger NETs enrichment in mitochondrial DNA of AOSD patients, highlighting IFN as a potential target for AOSD treatment [97].

## Conclusions

Neutrophilic dermatosis are a heterogeneous group of cutaneous disorders characterized by the histologic finding of a predominantly sterile neutrophilic infiltrate within the various layers of the skin, in the epidermal layer like pustular dermatoses, in the dermal layer like Sweet’s syndrome, in the hypodermal layer like pyoderma gangrenosum and in the overlapping layer like PAPA syndrome [98, 99]. All of which share a common pathological feature of non-infectious neutrophilic infiltration. While the clinical manifestations, laboratory indicators, and mechanisms of onset vary among this group of diseases, activated neutrophils play an important role in the pathogenesis of each. Research has found that the production of NETs by neutrophils may serve as a major form of neutrophil activation in the pathogenesis of NDs. This is demonstrated by the elevated expression levels of NETs in skin lesions and serum of NDs patients, enhanced ability of neutrophils from NDs patients to spontaneously produce NETs, and increased expression levels of upstream regulators of NETs production such as IL-36 and IFN. However, the regulatory mechanism of neutrophil production of NETs in these diseases lacks further investigation and exploration. In light of these findings, it is imperative to deepen our understanding of the mechanisms underlying neutrophil activation in NDs. Such an understanding may pave the way for the development of novel therapies that can effectively target the dysregulated immune response and promote better outcomes for patients affected by this group of disorders.
